# Dual-loaded nano pesticide system based on industrial grade scaleable carrier materials with combinatory efficacy and improved safety

**DOI:** 10.1186/s12951-024-02628-9

**Published:** 2024-06-20

**Authors:** Ningjun Li, Jianxia Cui, Jianjiang Zhao, Changcheng An, Zheng Wei, Yue Shen, Changjiao Sun, Chong Wang, Shenshan Zhan, Xingye Li, Anqi Wang, Dan Luo, Yan Wang

**Affiliations:** 1 Institute of Environment and Sustainable Development in Agriculture, Chinese Academy of Agricultural Sciences, Beijing, 100081 China; 2 Institute of Plant Protection, Hebei Academy of Agricultural and Forestry Sciences, Baoding, 071000 China; 3 Department of Biological and Environmental Engineering, Cornell University, Ithaca, NY 14853 USA

**Keywords:** Dual-loaded nano pesticide, Industrial scaleable system, Enhanced release property, Combinatory efficacy, Improved safety

## Abstract

Repeated and widespread use of single chemical pesticides raises concerns about efficiency and safety, developing multi-component synergistic pesticides provides a new route for efficient control of diseases. Most commercial compound formulations are open systems with non-adjustable released rates, resulting in a high frequency of applications. Meanwhile, although nano pesticide delivery systems constructed with different carrier materials have been extensively studied, realizing their actual scale-up production still has important practical significance due to the large-scale field application. In this study, a boscalid and pyraclostrobin dual-loaded nano pesticide system (BPDN) was constructed with industrial-grade carrier materials to facilitate the realization of large-scale production. The optimal industrial-scale preparation mechanism of BPDN was studied with surfactants as key factors. When agricultural emulsifier No.600 and polycarboxylate are used as the ratio of 1:2 in the preparation process, the BPDN has a spherical structure with an average size of 270 nm and exhibits superior physical stability. Compared with commercial formulation, BPDN maintains rate-stabilized release up to 5 times longer, exhibits better dispersion and spreading performance on foliar, has more than 20% higher deposition amounts, and reduces loss. A single application of BPDN could efficiently control tomato gray mold during the growing period of tomatoes due to extended duration and combinatory effectiveness, reducing two application times and labor costs. Toxicology tests on various objects systematically demonstrated that BPDN has improved safety for HepG2 cells, and nontarget organism earthworms. This research provides insight into creating safe, efficient, and environmentally friendly pesticide production to reduce manual operation times and labor costs. Accompanied by production strategies that can be easily scaled up industrially, this contributes to the efficient use of resources for sustainable agriculture.

## Introduction

Pesticides are an important material basis for defense against major agricultural pests and diseases and for guaranteeing food security and agricultural production. The world consumes 3.54 million tons of pesticide active ingredients every year, at present, the effectiveness and safety of pesticides need to be further improved, attributed to the wind, sun, rain, and pest resistance [1–3]. Declining utilization of pesticides results in increasing amounts of pesticide formulations and crop losses [4–6], which poses a challenge to the sustainable control of pests and pathogens [7, 8]. However, the use of chemical pesticides is one of the most effective control methods for most plant diseases and has provided irreplaceable benefits to crop production. Thus, improving the effectiveness and safety of chemical pesticides is crucial for efficient plant pest control. Based on long experience with pest control, one of the strategies usually recommended to improve utilization is the use of pesticide mixtures [9, 10] to reduce unnecessary large-scale and repeated pesticide applications [9, 11, 12]. The development of multi-component pesticide formulations provides a new route for effective control of diseases and pests. Alternatively, the development and application of compatible, synergistic-release model pesticide formulations containing different pesticides will be more efficient in accurately controlling pests and diseases and is one of the new challenges for chemical pesticides [13, 14].

The development of nanomaterials and nanotechnology provides new insights for the construction of efficient and safe pesticide delivery systems. Nanomaterials with small size effect, large specific surface area, and modifiability can improve the adhesion properties of pesticides on leaf surfaces and harmful target surfaces, thus improving efficiency, reducing the waste of formulations caused by coarse particles and imprecise release, and enhancing their biological activity and efficacy [15–17]. Alternatively, nanotechnology can enable penetration and accurate delivery of active ingredients to the target [15, 18], avoiding decomposition deactivation during delivery and prolonging the time that active ingredients are maintained within the effective control dose threshold [19–22], has the potential to improve the precision, targeting, and efficiency of pesticide formulations [23]. The properties and effects of nano-pesticide delivery systems constructed with different structures and materials have been extensively studied [24–28] and promoted the research of nano-pesticides to become the current research hotspot.

Gray mold, caused by *Botrytis cinerea Pers.*, occurs on more than 200 plant species, including tomato, and causes serious economic loss [29, 30]. For the management of gray mold, chemical control is one of the most effective and economical methods [31, 32]. However, the pesticide resistance of *Botrytis cinerea Pers.* is particularly severe [33, 34], and the selection of suitable chemicals for effective control is necessary to safeguard production of an abundance of plant crops. Boscalid is a carboxamide fungicide that can be used on a wide range of plants, including tomato, wheat, oats, and barley [35]. Boscalid, however, differs from some other carboxamide fungicides in its mode and site of action due to the inhibition of quinine reduction activity [36]. and studies have shown that boscalid possesses good biological activity against grey mold [37–39]. Additionally, quinone outside inhibitor fungicides including pyraclostrobin have also been similarly applied to control gray mold, but the risk for development of resistance to this kind of fungicide is high [40–42]. Recent research has shown that a mixture of pyraclostrobin and boscalid can act at two different positions in the electron transport chain to both improve efficacy and reduce the risk of resistance when pyraclostrobin is used alone [42, 43].

Currently, the repeated and amounts use of fungicides has accelerated the development of pesticide resistance. The pesticide compounding technology to control the same disease prevention and control of the two active ingredients in proportion to the mixture can achieve the purpose of efficiency, slow down the resistance, and extend the duration of existing pesticides. As a result, compounded formulations are gradually becoming more widely used. However, most of the compounded formulations currently are open systems that lack regulation of the release of active ingredients and prolongation of the duration, thus the combinatory control effect needs to be further improved. Furthermore, the large-scale application of novel formulations especially the nano pesticide formulations remains a technical challenge, especially the use of some industrially scalable carrier materials and technologies that facilitate industrial production. Realizing the actual scale-up production of pesticides based on the carrier system still has important practical significance, attributed to the pesticide formulation being a product of large-scale application in the field.

**Fig. 1:**
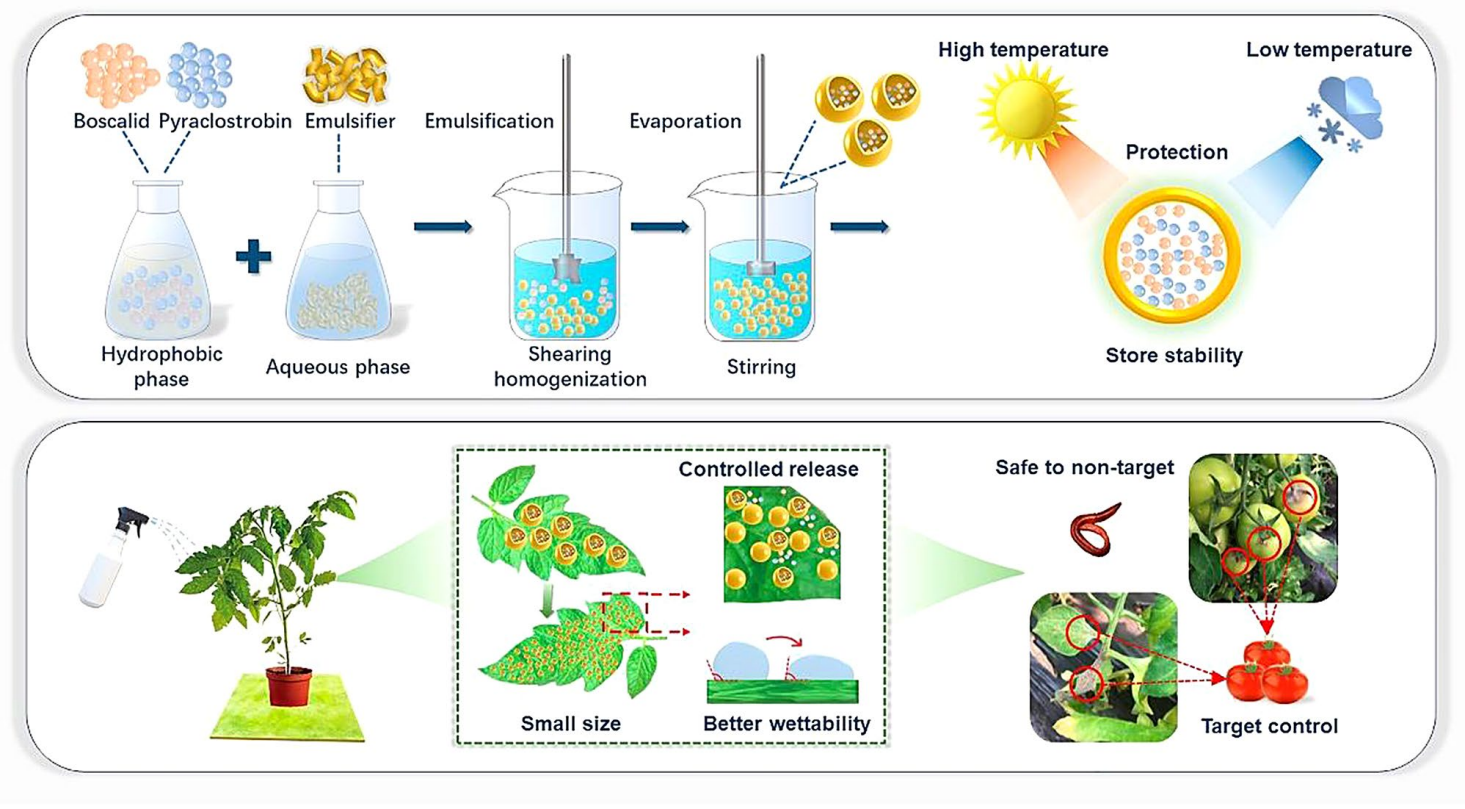
The schematic of BPDN

In this work, the dual-loaded nano pesticide system was constructed of pesticide synergistic combinations of pyraclostrobin and boscalid with industrial-grade scaleable carrier material for the effective prevention and control of gray mold, a major disease in the world. During the construction of the system, the type and content ratio of the surfactant were used as key factors to investigate the optimal industrial-scale preparation process of the boscalid and pyraclostrobin dual-loaded nano system (BPDN). The nano pesticide system has a spherical structure and exhibits good dispersion, deposition, and spreading performance on foliar with an average size of 270 nm. Bioassay and field efficacy tests demonstrated that a single application of the dual-loaded nano pesticide system could achieve effective control during the growing period of tomato, accompanied by the slow-release characteristics and extended duration, combinatory effectiveness. Toxicology tests on various objects systematically demonstrated that BPDN has improved safety for HepG2 cells in vitro, and nontarget organism earthworms. This research provides insight into creating safe, efficient, and environmentally friendly pesticide production to reduce manual operation times and labor costs. Accompanied by production strategies that can be easily scaled up industrially, this contributes to the efficient use of resources for sustainable agriculture. (Fig. 1)

## Materials and methods

### Materials

Technical boscalid (95%) was obtained from Beijing Jinyue Biotechnology Co., Ltd. Technical pyraclostrobin (96%) was purchased from Beijing Jinyue Biopharmaceutical Co., Ltd. Industrial-grade Polylactide (PLA, mw-100 kDa) was obtained from Nature Works. Co., Ltd. Polyvinyl alcohol (PVA, 87- 90%, Mw: 30,000–70,000) was supplied by Sigma-Aldrich (Shanghai) Trading Co., Ltd. Agricultural emulsifier No.600 and Agricultural emulsifier No.700 were supplied by Cangzhou Hongyuan agrochemical Co., Ltd. Polycarboxylate (PC) was purchased from Jiangsu Qingyu Chemical Technology Co., Ltd. Tween-85 was manufactured by Beijing Ruixinnuo Biotechnology Co., Ltd. Dichloromethane (analytical purity) was obtained from Sinopharm Chemical Reagent Co., Ltd. Methanol (chromatographic grade) and acetonitrile (chromatographic grade) were obtained from Thermo Fisher Scientific Co., Ltd. The deionized water used in the study was purified by Elix Essential 5 water purifier (Millipore, Massachusetts, USA). Tomato plants are grown in laboratory incubators. HepG2 cells were purchased from iCell Bioscience Inc., Shanghai. The test earthworms (Eisenia williamsii), purchased from East China Breeding Base in Jinan, Shandong Province.

### System construction

Among the different materials used to encapsulate pesticide active ingredients, industrial-grade polylactide (PLA) was chosen for the preparation of BPDN due to its safety, degradability, industrial-scale scalable, and low cost. The dual-loaded nanospheres were prepared by solvent evaporation combined with high-pressure homogenization technology [44, 45]. Boscalid and pyraclostrobin were initially dissolved in dichloromethane containing 3% PLA to form the hydrophobic phase. The emulsifiers were then dissolved in water to form the 1% emulsifiers containing aqueous phase. The hydrophobic phase was dispersed in the aqueous phase using a high-shear emulsifying machine (ATS, NANOJH10, Virgin Islands, British) to form the coarse emulsion. The coarse emulsion was then transferred to a high-pressure homogenizer (ATS, AH-100D, Virgin Islands, British) and treated at 250, 450, and 650 kPa three times to obtain a uniform emulsion.

The obtained fine emulsion was stirred for 20 h at room temperature at 600 rpm to eliminate excess organic solvents using a cantilever stirrer (IKA, EUROSTAR 60, Staufen, Germany). The nano-capsules were centrifuged at room temperature at 10,000 rpm for 10 min using a high-speed freezing centrifuge (Thermo Scientific, ST16R, Waltham, U.S.). The supernatant was then removed, and the precipitate was resuspended in deionized water. The process was repeated to remove excess impurities. The precipitates were then collected and frozen in an ultra-low temperature refrigerator (Haier, DW-86W100, Qingdao, China) and lyophilized in a freeze dryer (Boyikang, FD-1 A-50, Beijing, China) to remove excess water.

### The influence of surfactant on particle size and dispersibility of BPDN

In this work, we separately studied the effects of single surfactant and complex surfactants on the delivery system. At first, the influence of five surfactants, including pesticide emulsifier 600#, pesticide emulsifier 700#, polyvinyl alcohol (PVA), tween-85, and polycarboxylate (PC), on the particle size and dispersibility of BPDN were investigated. Pyraclostrobin and boscalid were dissolved in dichloromethane and PLA was added to obtain the hydrophobic phase. The five kinds of single surfactants () were dissolved in deionized water to obtain the aqueous phase. The hydrophobic phase was slowly added to the aqueous phase to prepare a coarse emulsion using a high-shear emulsifying machine (ATS, NANOJH10, Virgin Islands, British). The coarse emulsion was then transferred to a high-pressure homogenizer (ATS, AH-100D, Virgin Islands, British) to obtain a fine emulsion. Then, the BPDN solid powder was obtained by stirring evaporation, centrifugation, and freeze-drying.

Moreover, we mixed the two selected surfactants that demonstrated favorable effects on the particle size and dispersibility in the last study in different ratios, including 1:1, 2:1, 1:2, 3:1, and 1:3. Different BPDNs with different proportions of complex surfactants were prepared and their particle size and PDI were determined.

### Characterization

The size, PDI, and zeta potential of BPDN were measured using a Malvern laser particle size analyzer (ZETASIZER NANO, ZS90, Malvern Panalytical, British). The morphology of BPDNs was observed via scanning electron microscopy (SEM, SU8010, Hitachi Limited, Tokyo) and transmission electron microscopy (TEM, HT7700, Hitachi Limited, Tokyo). The samples were deposited on a silicon wafer and copper mesh for scanning electron microscopy and transmission electron microscope observation, respectively.

Different samples were taken and pressed separately. Then IR spectra were recorded using a Fourier transform infrared spectrometer (Nicolet6700, Thermo Nicolet Corporation, USA).

### Controlled-release properties

To evaluate the in vitro release of the nano delivery system, the cumulative release of the two components, boscalid and pyraclostrobin, was determined by dialysis at different times. The assay procedure consisted of three different sets of samples, including BPDN, BPWDG, and the technical pesticide compound of boscalid and pyraclostrobin mixed in a 2:1 ratio, which were packed into dialysis bags and shaken in a shaker at 25 °C using methanol as a medium. 5 ml of aliquots was removed at specific time intervals and 5 ml of the buffer solution was replenished in the system simultaneously to compensate for the removed aliquots. The concentration of the active ingredient was determined by high-performance liquid chromatography (HPLC) and the cumulative release was calculated as follows:

$${\rm{Cumulative}}\,{\rm{relative}}\,{\rm{percentage}} = {{{V_0} \times {C_T} + V \times \sum\nolimits_{n = 1}^{T - 1} C } \over W} \times 100\%$$

Where V0 represents the volume of the release solution; CT represents the mass concentration of the active ingredient in the dissolution medium in milligrams per milliliter (mg/mL) measured at the release time point; V represents the volume of each sample in milliliters (mL); W represents the total amount of active ingredients loaded in milligrams (mg).

### Stability test

The prepared samples were set in three groups then packed and stored in the sealed tube. According to the Collaborative International Pesticides Analytic Council general methods (CIPAC MT 46) and Chinese storage stability testing standards (GB/T 19136 − 2003, NY/T 1427–2016, GB/T 19137 − 2003), the three groups were stored at 0 ± 2 ℃ for 7 days, 25 ± 2 ℃ for 14 days, and 54 ± 2 ℃ for 14 days. Then the stability assessment was carried out at fixed time intervals. The particle size and PDI were measured to evaluate physical storage stability. At the same time, the morphology and internal structure of BPDNs were characterized by transmission electron microscope (TEM, HT7700, Hitachi Limited, Tokyo).

### Foliar performance and retention

In this work, we comprehensively demonstrate the behavioral characteristics of the nanocarrier system on the leaf surface and elaborate on its relevance to combinatory effects through the study of foliar retention, wetting performance, and dispersion properties.

At first, fresh cucumber and cabbage leaves were collected, and uniform-size foliar samples were prepared with a regular puncher. Leaf area “S” (cm2) was measured with a portable leaf area measuring instrument. Fine-tipped tweezers were placed in the liquid pesticides, and the mass was recorded as “M0” on the electronic balance with an accuracy to 0.001 g. The leaves were then completely immersed in the liquid pesticide for 20 s, and suspended vertically for 30 s after removal from the liquid pesticide. When there was no more dripping, the mass of tweezers and solution was recorded as “M1”. Deionized water was used as a blank control, and boscalid/pyraclostrobin water dispersible granules (BPWDG) were used as the control group. Each group was repeated 5 times independently according to the field spraying concentration of commercial formulations. The foliar retention (R) was calculated according to Equation as follows:

$${\rm{R }}\left( {{\rm{mg/c}}{{\rm{m}}^{\rm{2}}}} \right){\rm{ = }}\left( {{{\rm{M}}_{\rm{0}}}{\rm{ - }}{{\rm{M}}_{\rm{1}}}} \right){\rm{ /S}}$$

The contact angle of BPDN was determined using deionized water, commercially available formulations water water-dispersible granule 1(WDG1) and water-dispersible granule 2 (WDG2) as controls, and the leaves of the target plant tomato as the foliage to be tested. The concentrations of the pesticide formulations to be tested were set based on the recommended doses in the field for the commercially available formulations.The leaves fixed flat on the slides were placed on the carrier table of the leaf surface contact angle measuring instrument for contact angle determination. The contact angle was measured using the five-point fitting method, and each sample was measured five times to obtain the mean and standard deviation.

Moreover, the above three sets of samples were sprayed with a spray gun to the clean tomato leaves with the same concentration of active ingredients in each group. The leaves of the sprayed samples were allowed to dry naturally and observed using ESEM to study the dispersion performance.

### Bioassays

The initial dose of the experiment was set according to the recommended dose of the Pesticide Information Registry Network, and then four concentrations were set up with this as the intermediate concentration. The boscalid/pyraclostrobin dual-loaded nanospheres were dispersed in four concentration gradients (380 mg/L, 760 mg/L, 1,140 mg/L, and 1,520 mg/L). BPWDG was used as the control group. The solution was then evenly sprayed on the tomato leaves. After 3 and 6 days, the tomato leaves were collected, with the back facing up and the petiole wrapped with absorbent cotton to maintain adequate moisture. The tomato leaves were placed in a 150 mm Petri dish covered with wet filter paper, and each leaflet was inoculated with a cake of *Botrytis cinerea* with a diameter of 5 mm. The relative control effect was calculated by measuring the diameter of the lesion with the cross method after 2 days of 24 ℃ light incubation.

### Field control effect

The experiment was carried out according to the guidelines for field efficacy trials of Fungicides against *Botrytis cinerea* (GB / T 17980.28–2000). The experiment was carried out in a solar greenhouse, and Provence tomatoes were used. The control treatment dose was set according to the recommended dose of the Pesticide Information Registry Network, and then experimental concentrations were set based on this. There were five treatments, including three concentration gradients (380 mg/L, 760 mg/L, and 1,140 mg/L) in the BPDN group, 380 mg/L in the BPWDG group, and a blank control group. The area of each treatment was 22 m2, and the dosage of each treatment was 2 L (900 L per hectare). When *Botrytis cinerea* occurred sporadically, BPDN was applied only once, and BPWDG was applied three times at a specific time interval. At different time intervals after application, the incidence of disease was investigated, the number of diseased leaves and fruits were recorded, and the control effect was calculated. The control effect results of three applications of the commercially available formulations were strictly compared with BPDN at the same dose of 1140 mg/L while maintaining the same dosage.

### Cytotoxicity

The CCK-8 assay was used to determine the effect of boscalid/pyraclostrobin dual-loaded nanoformulations on the cell viability of HepG2 cells [46]. HepG2 cells at the logarithmic growth stage were seeded onto 96-well microtiter plates with 5% CO2 and incubated overnight at 37℃ in a constant temperature incubator. The boscalid/pyraclostrobin dual-loaded nanoformulation and the control samples were set up as five different concentrations (5, 25, 50, 75, 90 mg/L), 100 µL was added to each treatment, and three replicates of each treatment were performed, and kept for 24 h. Then the culture medium was removed, each well was washed three times with PBS, and the culture medium containing 10% CCK-8 was added according to 100 µL/well in. After incubation for 2 h in 5% CO2 and 37℃ in a constant temperature incubator, the absorbance values at 450 nm were monitored with an enzyme marker. The absorbance values of each group were entered into Excel and the relative viability was calculated as follows:

Relative viability % = (OD value of test group - background OD value) / (mean OD value of control group - background OD value) × 100%.

Where the background OD value is the absorbance of adding CCK-8 reagent and medium only.

### Acute toxicity to earth worms

Pesticide solutions of the BPDN, BPWDG, and the mixture of technical pesticides of boscalid and pyraclostrobin were prepared at six different concentrations respectively, including 1200 mg/L, 1000 mg/L, 500 mg/L, 250 mg/L, 150 mg/L, and 50 mg/L. The healthy earthworms were introduced into culture dishes, and then the pesticide samples were added with 1 ml per dish. The survival rate of the earthworms for each treatment was assessed after 24 h and 48 h.

## Results and discussion

### The influence of single surfactant on particle size and dispersibility of BPDN

**Fig. 2:**
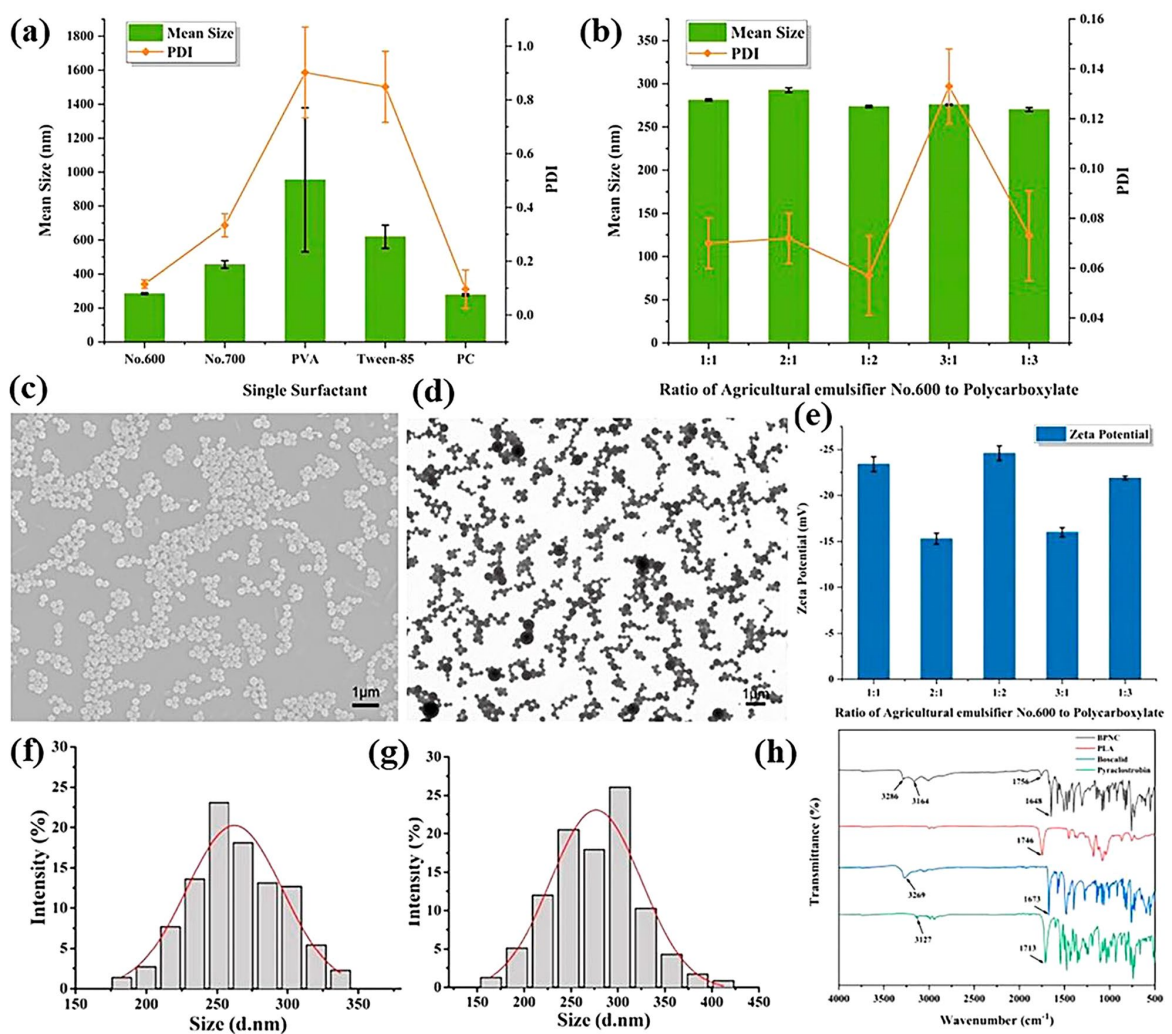
The Preparation and technical optimization of BPDN. (**a**) The influence of single surfactant on particle size and dispersibility of BPDN. (**b**) The influence of complex surfactants on particle size and dispersibility of BPDN. (**c**) SEM. (**d**) TEM. (**e**) The influence of complex surfactants on zeta potential of BPDN. (**f**)statistical particle size of BPDN based on SEM. (**g**) statistical particle size of BPDN based on TEM. (**h**)FT-IR spectroscopy of BPDN

The average particle size and PDI are important indexes to evaluate the nano pesticide loading system. The five surfactants, agricultural emulsifier No.600, agricultural emulsifier No. 700, PVA, tween-85, and PC, were studied by single factor experiments. The average particle size of BPDN prepared with agricultural emulsifier No. 700, PVA or tween-85 was more than 300 nm, while that of BPDN prepared with agricultural emulsifier No. 600 and PC was lower than 300 nm. A PDI of more than 0.3 indicates that the particle size is not uniform [47, 48]. The measured PDI of BPDN prepared by agricultural emulsifier No. 700, PVA, and tween-85 was more than 0.3, especially for PVA and tween-85, which were much greater than 0.5. This result further indicated that the particle size was not uniform, while the PDI of nanoparticles prepared by agricultural emulsifier No. 600 and PC was about 0.1. Based on the average particle size and PDI, agricultural emulsifier No. 600 and PC were selected as the complex surfactants for the preparation of BPDN. (Fig. 2)

### The influence of complex surfactants on particle size and dispersibility

The effects of surfactant ratio on particle size and PDI of BPDN were investigated by mixing agricultural emulsifier No.600 and PC at different ratios (1:1, 2:1, 1:2, 3:1, and 1:3). The particle size and PDI of BPDN prepared by five complex surfactants of different ratios are all below 300 nm, which indicate that BPDN with small particle size and uniform dispersion can be prepared using different surfactant ratios. The zeta potential of BPDN was positively related to the proportion of anionic surfactant PC. When the absolute value of the zeta potential was greater than 30 mV, the electrostatic repulsion between BPDNs was significant, resulting in superior physical stability. When the ratio was 1:2, the particle size of BPDN was 273.8 nm, the PDI was 0.057, and the absolute value of the zeta potential was 24.6 mV.

Agricultural emulsifier No.600 is a non-ionic surfactant, which can adhere to the surface of nanoparticles to form an adsorption layer. The resulting layer produces steric effects, which can prevent the aggregation of nanocapsules. PC, an anionic surfactant, can provide enough charge to maintain the stability of nanocapsules by electrostatic repulsion. When the ratio was 1:2, the nanosystem can play the best combination effect of the layer producing steric effects of Agricultural emulsifier No.600 and the electrostatic repulsion effect of PC, which can make the nanocapsules have good dispersion and stability. Therefore, when the ratio was 1:2, the particle size of BPDNs was small, the distribution was uniform and stable, and the absolute value of zeta potential was the largest, resulting in superior physical stability. Therefore, the optimal ratio of agricultural emulsifier No.600: PC was 1:2.

### Morphology and physicochemical characteristics

The morphology and internal structure of BPDN were characterized by scanning electron microscopy (SEM) and transmission electron microscopy (TEM). BPDNs prepared with agricultural emulsifier No. 600 and Polycarboxylate as surfactants were spherical with a smooth appearance and regular morphology. The size distribution of the nanoparticles was uniform, and there was no agglomeration between the particles. The average particle size of BPDN was 270 nm, which was consistent with the hydrated particle size measured by dynamic light scattering. The results demonstrate that the prepared dual-delivery system has the characteristics of small particle size, uniform dispersion, and regular morphology, which can improve physical stability. This is conducive to the small size effect and interfacial effect, which facilitate to improvement of the contact area and dispersion performance of the formulations on the foliar surface, further enhancing the adhesion performance, and reducing the spraying loss in the application process.

The Fourier transform infrared (FTIR) spectrum of boscalid shows a broad characteristic peak at approximately 3269 cm − 1, which was assigned to the telescopic vibration of the N-H bond. And a sharp peak at 1673 cm-1, which is a telescopic vibration peak assigned to the C = O bond. For pyraclostrobin, the C-H stretching vibration peak on the benzene ring is at 3127 cm-1, and the carbonyl C = O stretching vibration peak is 1713 cm-1. Similarly, PLA is a C = O bond stretching vibration peak at 1746 cm-1. Comparing the IR absorption peaks of the BPDN with those of the above three compounds, it can be found that all four characteristic peaks of boscalid, pyraclostrobin, and PLA appear at the strong absorption peaks of BPDN. The results indicated that the two active ingredients were encapsulated in the PLA carriers, and were entangled into one by physical cross-linking.

### Release behavior

Individual release profiles of the two active ingredients showed that the release curves of boscalid and pyraclostrobin for each formulation showed a similar direction and pattern, implying that the two active ingredients were released synergistically in the dual-loaded system. The release behavior of the total active ingredient showed that the release rate of BPWDG had reached 70.12% at 36 h and only released 5.34% of the total at 36–240 h, which is at a very low dose release level. The technical pesticide compound had released 62.91% at 60 h, with a sharp decrease in the release speed in the subsequent 60–240 h. BPDN, on the other hand, first went through a rapid release phase of 1–10 h during the whole 240 h of the test, and then gradually released at a steady rate, and was still able to maintain a stable release rate and prolonged the release period at 240 h.

The results indicate that compared to BPWDG and the technical pesticide, BPDN can significantly prolong the release time and maintain a stable release rate to achieve long-term stable release among the plant growth cycle and disease incidence cycle, due to the encapsulation of the carrier materials. This contributes to the prolongation of the holding period, and the achievement of effective control of pests and diseases with a single application covering the entire life cycle, which can be further verified by field efficacy.

### Stability

The three groups of samples were stored at 0 ℃ for 7 days, and 25 ℃ and 54 ℃ for 14 days, and the particle size, PDI, and morphology of samples were investigated. From the observation of the appearance, there was no significant physical change in each sample after storage, and no precipitation visible to the naked eye. Only after 14 days of storage at 54 °C, the color of the solution slightly deepened, probably related to the evaporation of water.

**Fig. 3:**
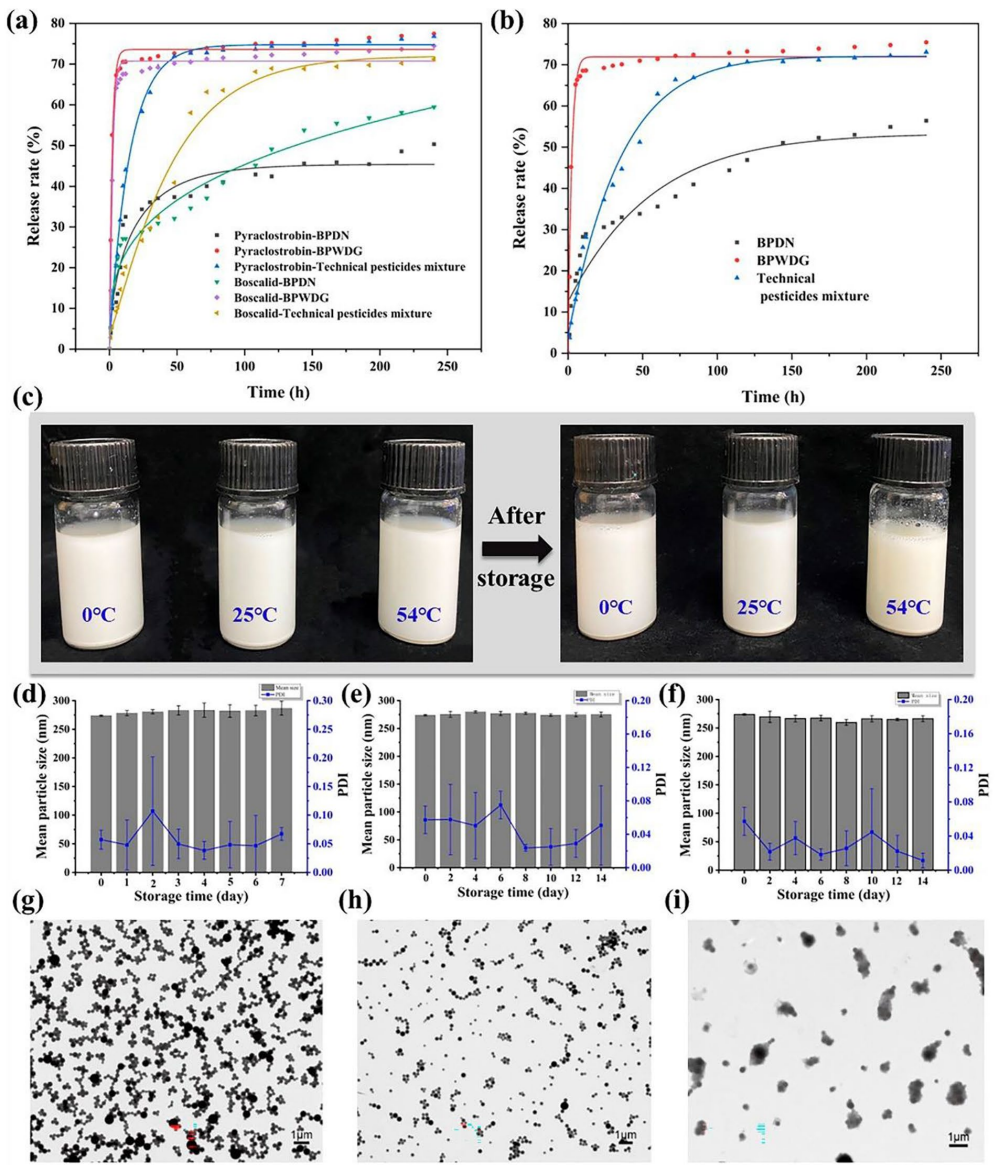
(**a**)Release fitting curve of two different active ingredients of different formulations. (**b**) Release fitting curve of total amounts of active ingredients. (**c**) Observation of appearance under different temperature storage. Mean particle size and PDI of BPDN under (**d**) 0 °C. (**e**) 25 °C. (**f**) 54 °C. (**g**) TEM of BPDN under 0 °C. (**h**) TEM of BPDN under 25 °C; (**i**) TEM of BPDN under 54 °C

The average particle size of BPDNs remained stable at 0 ℃, 25 ℃, and 54 ℃ without significant change. The PDI of samples was less than 0.3 after storage at different temperatures. The morphology of BPDNs after storage was determined by transmission electron microscopy (TEM). At 0 ℃ and 25 ℃, the morphology of BPDNs was uniform and regular, with good stability; the morphology of BPDNs at 54 ℃ was fuzzy, which may be caused by the fact that the storage temperature of 54 ℃ approached the glass transition temperature of PLA; the glass transition process of PLA results in a change of shape of PLA from rigid to flexible, and the morphology of BPDNs changed due to this transition [49, 50]. However, BPDNs still showed good dispersion without remarkable particle agglomeration after storage at 54 ℃ for 14 days. The results reveal that the industrial-grade carrier material that we used in this work has good stability under different temperature storage conditions, meanwhile, the carrier material plays a protection effect on active ingredients by encapsulation to avoid the degradation of active ingredients at different temperatures. (Fig. 3).

### Foliar retention

**Fig. 4:**
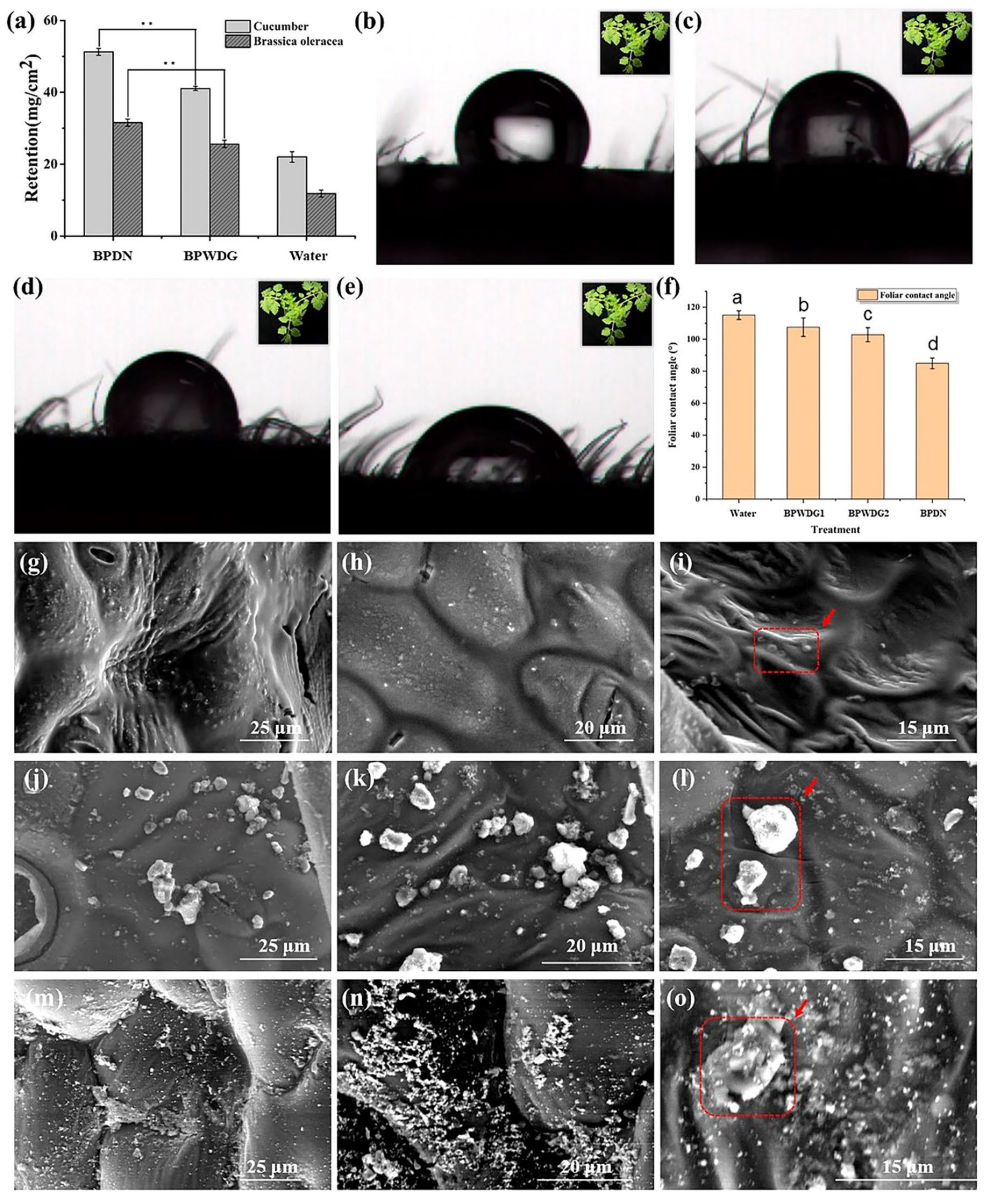
The foliar performance of BPDN. (**a**) The retention volumes on the surfaces of cucumber and cabbage leaves. (one-way ANOVA, LSD test, ** *P* < 0.01) (**b**) Contact angle of water on the leaf surface of tomato leaves. (**c**) Contact angle of WDG1 on the leaf surface of tomato leaves (**d**) Contact angle of WDG2 on the leaf surface of tomato leaves. (**e**) Contact angle of BPDN on the leaf surface of tomato leaves. (**f**) Analysis of contact angle. (one-way ANOVA, Duncan test, *p* < 0.05) (**g**-**i**): ESEM images of BPDN. (**j**-**l**) ESEM images of WDG1. (**m**-**o**) ESEM images of WDG2

Increasing the retention of pesticides on crop leaves is a key factor for improving the effective utilization of pesticides in target pests. The retention of BPDN and BPWDG on cucumber and cabbage leaves was determined by the immersion weighing method, and the results are shown in Fig. 4a. Compared with BPWDG, the retention of BPDN on cucumber leaves (51.3 ± 0.9 mg/cm^2^) was improved about 25%. Due to the wax layer on the surface of cabbage leaves, the retentions of two formulations are lower than that on cucumber leaves, but BPDN still can improve about 23% retention amounts when compared with the retention of BPWDG (25.6 ± 0.9 mg/cm^2^). One-way ANOVA analysis indicated that retention of BPDN on cucumber and cabbage leaves was significantly improved compared with that of BPWDG. The prepared nanocapsules’ increased foliar retention may be due to their smaller size effect, the larger contact area with foliage, and can facilitate to the reduction of the drift loss during the application.

### Foliar wetting and dispersing properties

In this work, the contact angle of BPDN on tomato leaves was determined with deionized water and commercially available formulations WDG1 and WDG2 as controls. The experimental results showed that the contact angle of deionized water on tomato leaves was 115.1 ± 2.7°. Those of commercially available WDG1 and WDG2 on tomato leaves were 107.5 ± 5.8°, and 102.8 ± 4.4°, respectively, while the contact angle of the dual-loaded nano-formulations prepared in this study on tomato leaves was less than that of the water and commercially available formulations, which was 84.9 ± 3.4°. Significance analysis of the contact angle data for the four treatment groups showed that the contact angle on tomato leaves was reduced and significantly different for the BPDN compared to water. Compared with commercially available WDG1 and WDG2, the contact angle on tomato leaves was significantly reduced for the BPDN.

Meanwhile, ESEM images demonstrated that the two commercial WDGs showed an aggregated irregular distribution of large particles on the leaf surface. Compared with the control, BPDN showed a more uniform distribution, smaller adherent particles on the leaf surface, more easily embedded in the leaf surface structure, and stronger spreading ability. This is beneficial for improving the adhesion efficiency with the leaf surface and pesticide utilization efficiency.

Therefore, due to the small size, BPDN is conducive to adhering to the leaf surface in a larger area, thereby improving pesticide droplets’ dispersing, wetting, and retention properties on the target crop leaves. In addition, nanoparticles play an important effect in the energy dissipation increase and pesticide droplets’ rebound behavior control on the leaf surface during the spraying process, which is beneficial for improving spraying performance and reducing the contact angle on the leaf surface [51–53]. The improved foliar performance of BPDN is conducive to reducing the loss and drift during the delivery process, and improving the utilization of formulations. 

### Bioactivity

**Fig. 5:**
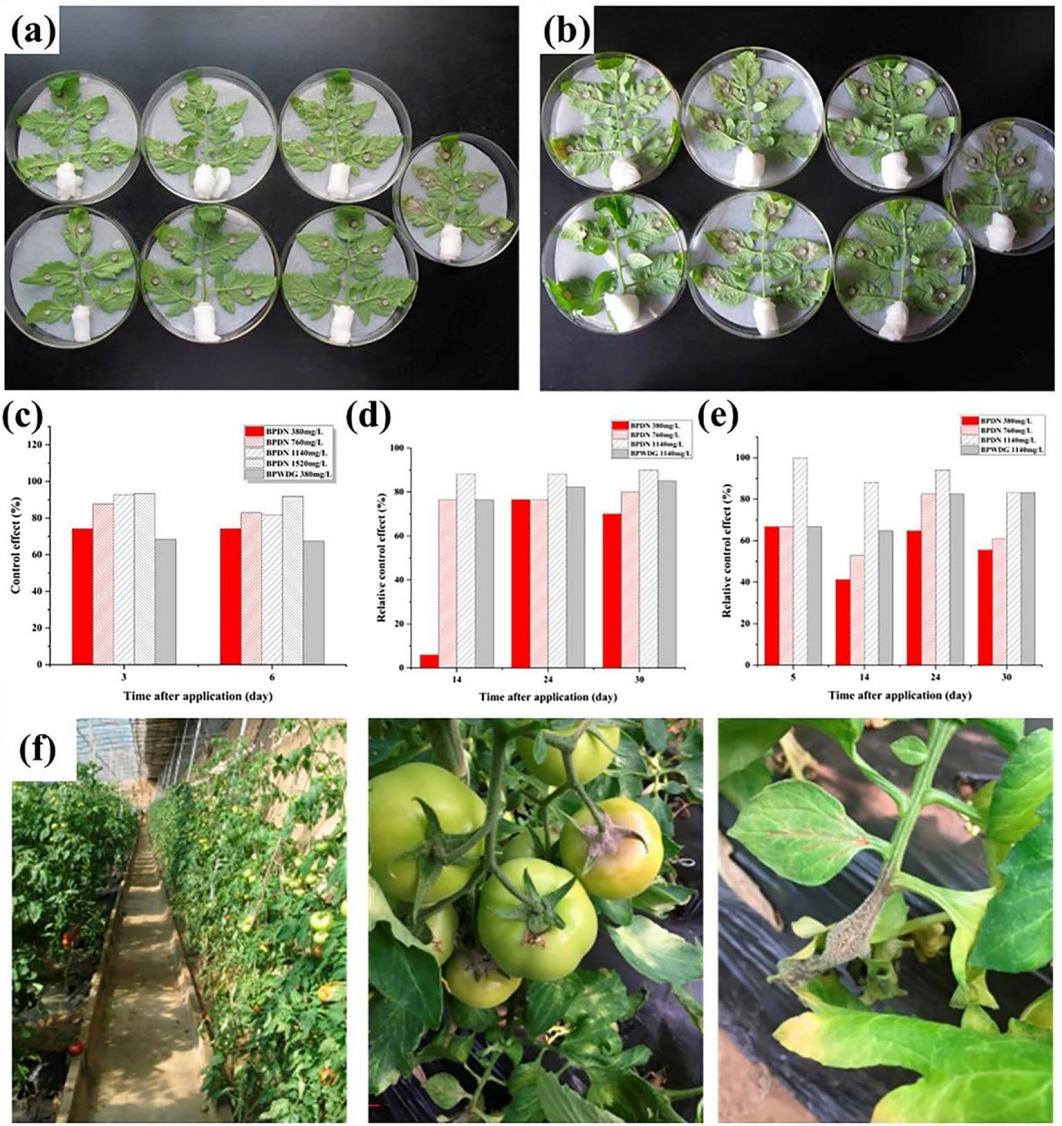
Growth status of *Botrytis cinereal* (**a**) 3 days after treatment. (**b**) 6 days after treatment. (**c**) The bioassays of BPDN on tomato gray mold. (**d**) Field control effect of tomato gray mold (leaves). (**e**) Field control effect of tomato gray mold (fruits). (**f**) Field investigation of tomato gray mold

The bioactivity of BPDN against *Botrytis cinerea* was determined with BPWDG used as the control treatment. The growth diagram of *Botrytis cinerea* is shown in Fig. 5. The control effect of BPDN against *Botrytis cinerea* can be maintained at a relatively stable level with the extension of time after treatment. Along with the increase in concentration from 380 mg/L to 1520 mg/L, the efficacy of BPDN increased to 93.41%, and after 6 days, the control effect could still be maintained at 91.8%, which indicated that a good control effect could be achieved against tomato gray mold fungus *Botrytis cinerea*. At 70% reduction compared with the market recommended field dose of 1260 mg/L, BPDN, and BPWDG demonstrated comparable control efficacy of 74.24% and 68.41%, respectively (after 3 days), and the efficacy was still maintained at this level after 6 days. Alternatively, at a 40% reduction compared with the marketed recommended field dose, BPDN was consistently above 80% and showed good persistence. The bioactivity results demonstrated that BPDN has a good control effect on tomato gray mold fungus *Botrytis cinerea*, and helps to reduce the application amounts and prolong the duration. This conclusion was further verified by the filed control test.

**Table 1:** Field control effect of tomato gray mold (leaves)

Formulations	Application amounts (mg/L)	Control effect (%)		
		14 days	24 days	30 days
BPNC	380	5.9	76.5	70.0
	760	76.5	76.5	80.0
	1140	88.2	88.2	90.0
BPWDG	380*3 = 1140	76.5	82.35	85.0

**Table 2:** Field control effect of tomato gray mold (fruits)

Formulations	Application amounts (mg/L)	Control effect (%)			
		5days	14 days	24 days	30 days
BPNC	380	66.7	41.2	64.7	55.6
	760	66.7	52.9	82.4	61.1
	1140	100	88.2	94.1	83.3
BPWDG	380*3 = 1140	66.7	64.7	82.4	83.3

### Field Control Effect

The efficacy of BPDN against the gray mold of tomatoes was investigated in the field (Table 1), (Table 2). Along with different application concentrations increasing from 380 mg/L to 1140 mg/L, the field control efficacy was improving. To maintain the uniformity of the same application dose and control comparison conditions, the efficacy of BPDN and commercial BPWDG was systematically investigated four times in 30 days. At 1140 mg/L (10% less than the commercially recommended dose), BPDN maintained 90% control after only one application for 30 days. Effective control was achieved with only one application during the tomato reproductive period, while at the same application dose, commercial BPWDG was 85% effective after 30 days accompanied by 3 applications. The results showed that BPDN can significantly reduce the times of applications, which is conducive to reducing the cost of labor operations.

### Safety evaluation

**Fig. 6:**
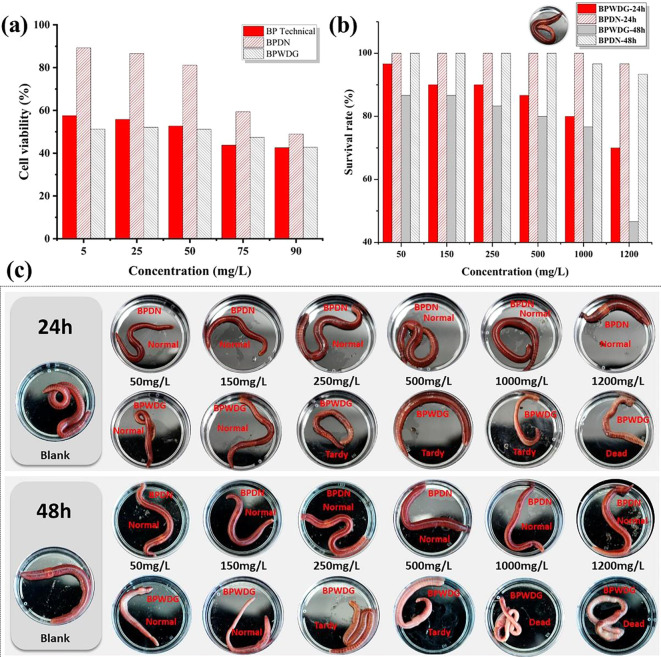
(**a**) cytotoxicity to HepG2 cells. (**b**-**c**) Acute toxicity to earthworms

The safety of pesticide formulations is always one of the most important factors affecting their application in the field. We systematically investigated the safety of multiple targets, including in vitro cellular and non-target organism earthworms (Fig. 6). Especially, earthworms are typical representatives of beneficial organisms designated in the toxicological evaluation criteria for pesticides. In vitro systematic evaluation of the cytotoxicity of BPDN in HepG2 cell culture was performed. The results demonstrated that Cell viability was consistently higher than 80% for BPDN treatment at concentrations of 50 mg/L and below, but less than 60% for cell viability under control treatments technical and commercial BPWDG. When the different pesticide formulations were treated at concentrations ranging from 5 mg/L to 90 mg/L, the cytotoxicity of BPDN was always lower than that of the BP technical and commercial BPWDG, which showed that BPDN has the potential to reduce toxicity.

The toxicity evaluation of different formulations in earthworms suggested that when the treated concentration was increased from 50 mg/L to 1200 mg/L, earthworms always maintained greater than 95.0% survival of BPDN treatment for 24 h and greater than 90.0% survival for 48 h. Furthermore, earthworms survived significantly better in the same concentration treatment of BPDN than in BPWDG. At a concentration of 250 mg/L, the earthworms treated with BPWDG were slowed down and even died, and the toxicity was significantly increased, while the earthworms treated with BPDN still maintained normal vital signs. When the concentration of the preparation reached 1200 mg/L, the survival rate of earthworms under the 48 h BPWDG treatment was 46.7%, whereas the survival rate of earthworms under the 48 h BPDN treatment was still as high as 93.3%, which was almost twice as much as that of the BPWDG treatment. The results verified that BPDN significantly reduced the toxicity to earthworms, which may be attributed to the encapsulation of biodegradable carriers and the absence of organic solvents, which effectively prevented the direct contact between pesticides and earthworms while improving environmental friendliness and safety. However, since these tests are still preliminary toxicity measurements, the safety determination needs more long-term and dynamic monitoring to further verify the possibility of BPDN in the practical application of more disease control.

## Conclusion

In this paper, we constructed a dual-loaded nano pesticide system with boscalid and pyraclostrobin as pesticide payloads and industrial-grade scalable materials as carrier, allowing for the realization of large-scale industry production and field application. The industrial-grade preparation mechanism of the BPDN was investigated with the surfactant type and mixing ratio as the key factors. Specifically, the surfactant composition differentially affects the size, particle distribution, and zeta potential of BPDN, and further influences the role of their nano effects. The BPDN was constructed through a simple and easier scale-up method with a spherical structure and an average particle size of 270 nm, which exhibits good storage stability. The BPDN showed improved dispersion, deposition, and spreading performance on foliar, which contributed to reduced roll-off and loss of formulation on the foliar surface. Alternatively, BPDN showed more adjustable release properties, and based on this, it is possible to realize a short time of sudden release, but also to maintain a longer period of slow release, prolonging the period of persistence. In addition, a single application of the dual-loaded nano pesticide system could achieve effective control of tomato gray mold during the growing period of tomato, compared with three times applications of commercial BPWDG, this is attributed to the extended duration and combinatory effectiveness of BPDN. Toxicology tests on various objects including in vitro HepG2 cells, and nontarget organism earthworms were systematically studied and verified that BPDN has reduced toxicity and improved safety. This work provides a promising approach to developing an industrial scaleable dual-functional nano pesticide system with more safe, efficient, and environmentally friendly performance, which is beneficial to reducing manual operation times and labor costs. Based on the production strategies that can be easily scaled up industrially, this work contributes to the scale-up promotion and application, further promoting the efficient use of agricultural resources and the development of sustainable agriculture.

## Data Availability

No datasets were generated or analysed during the current study.

## References

[1] Liang J, Tang S, Cheke RA (2016). Beverton–Holt discrete pest management models with pulsed chemical control and evolution of pesticide resistance. Commun Nonlinear Sci Numer Simul.

[2] Bass C, Jones CM (2018). Editorial overview: pests and resistance: resistance to pesticides in arthropod crop pests and disease vectors: mechanisms, models and tools. Curr Opin Insect Sci.

[3] Balabanidou V, Grigoraki L, Vontas J (2018). Insect cuticle: a critical determinant of insecticide resistance. Curr Opin Insect Sci.

[4] S.U. Karaağa, amp, Insecticide Resistance, World Health Organization (2012).

[5] Liang J, Tang S, Nieto JJ, Cheke RA (2013). Analytical methods for detecting pesticide switches with evolution of pesticide resistance. Math Biosci.

[6] Liang J, Tang S, Cheke RA (2016). Pure Bt-crop and mixed seed sowing strategies for optimal economic profit in the face of pest resistance to pesticides and Bt-corn. Appl Math Comput.

[7] Pavlidi N, Vontas J, Van Leeuwen T (2018). The role of glutathione S-transferases (GSTs) in insecticide resistance in crop pests and disease vectors. Curr Opin Insect Sci.

[8] Søgaard Jørgensen P, Folke C, Henriksson PJG, Malmros K, Troell M, Zorzet A (2020). Coevolutionary governance of antibiotic and Pesticide Resistance. Trends Ecol Evol.

[9] Delnat V, Tran TT, Janssens L, Stoks R (2019). Resistance to a chemical pesticide increases vulnerability to a biopesticide: effects on direct mortality and mortality by predation. Aquat Toxicol.

[10] Borel B. WHEN THE PESTICIDES RUN OUT, 543, 302–304 (2017).

[11] Wilkie MP, Hubert T, Boogaard M, Birceanu O. Control of Invasive Sea lampreys using the piscicides TFM and Niclosamide: Toxicology, successes & Future prospects. Aquat Toxicol. 2018;211. 10.1016/j.aquatox.2018.12.012. 10.1016/j.aquatox.2018.12.012 30770146

[12] Liang J, Tang S, Cheke RA, Wu J (2015). Models for determining how many natural enemies to release inoculatively in combinations of biological and chemical control with pesticide resistance. J Math Anal Appl.

[13] Ding F, Li L-X, Peng W, Peng Y-K, Liu B-Q (2019). Molecular basis for the resistance of American sloughgrass to aryloxyphenoxypropionic acid pesticides and its environmental relevance: a combined experimental and computational study. Chemosphere.

[14] Mdee LK, Masoko P, Eloff JN (2009). The activity of extracts of seven common invasive plant species on fungal phytopathogens. S Afr J Bot.

[15] Zhao H, Fau - X, Cui Y, Cui H, Auid, Wang C, Wang Y, Fau - Sun B, Sun C, Fau - Cui Z, Cui B, Fau - Zeng ZA-O, Zeng. Development Strategies and Prospects of Nano-based Smart Pesticide Formulation, (1520–5118 (Electronic)). 10.1021/acs.jafc.7b02004 28654254

[16] Kumar S, Nehra M, Dilbaghi N, Marrazza G, Hassan AA, Kim K-H (2019). Nano-based smart pesticide formulations: emerging opportunities for agriculture. J Control Release.

[17] Nuruzzaman M, Rahman MM, Liu Y, Naidu R (2016). Nanoencapsulation, Nano-guard for pesticides: a new window for safe application. J Agric Food Chem.

[18] Nair R, Varghese SH, Nair BG, Maekawa T, Yoshida Y, Kumar DS (2010). Nanoparticulate material delivery to plants. Plant Sci.

[19] Wang A, Cui J, Wang Y, Zhu H, Li N, Wang C, Shen Y, Liu P, Cui B, Sun C, Wang C, Gao F, Zeng Z, Cui H (2020). Preparation and characterization of a Novel controlled-release Nano-Delivery System loaded with pyraclostrobin via high-pressure homogenization, Pest Manag. Sci.

[20] Wang A, Wang Y, Sun C, Wang C, Cui B, Zhao X, Zeng Z, Yao J, Yang D, Liu G, Cui H (2018). Fabrication, characterization, and Biological Activity of Avermectin Nano-delivery systems with different particle sizes. Nanoscale Res Lett.

[21] Wang Y, Wang A, Wang C, Cui B, Sun C, Zhao X, Zeng Z, Shen Y, Gao F, Liu G, Cui H. Synthesis and characterization of emamectin-benzoate slow-release microspheres with different surfactants, (2045–2322 (Electronic)). 10.1038/s41598-017-12724-6 PMC5630577 28986529

[22] Zhu H, Shen Y, Cui J, wang A, Li N, Wang C, Cui B, Sun C, Zhao X, Wang C, Gao F, Zhan S, Guo L, Zhang L, Zeng Z, Wang Y, Cui H (2020). Avermectin loaded carboxymethyl cellulose nanoparticles with stimuli-responsive and controlled release properties. Ind Crop Prod.

[23] Kah M, Hofmann T (2014). Nanopesticide research: current trends and future priorities. Environ Int.

[24] Elsharkawy MM, Derbalah A (2019). Antiviral activity of titanium dioxide nanostructures as a control strategy for broad bean strain virus in faba bean. Pest Manag Sci.

[25] Malandrakis AA, Kavroulakis N, Chrysikopoulos CV (2020). Use of silver nanoparticles to counter fungicide-resistance in Monilinia fructicola, Sci. Total Environ.

[26] Elsharkawy MA-OX. Induced systemic resistance against Cucumber mosaic virus by Phoma sp. GS8-2 stimulates transcription of pathogenesis-related genes in Arabidopsis, (1526–4998 (Electronic)). 10.1002/ps.5193 30168656

[27] Derbalah A, Elsharkawy MM, Hamza A, El-Shaer A (2019). Resistance induction in cucumber and direct antifungal activity of zirconium oxide nanoparticles against Rhizoctonia solani. Pestic Biochem Phys.

[28] Derbalah A, Shenashen M, Hamza A, Mohamed A, Safty SE (2018). Antifungal activity of fabricated mesoporous silica nanoparticles against early blight of tomato. Egypt J Basic Appl Sci.

[29] Li T, Li H, Liu T, Zhu J, Zhang L, Mu W, Liu F (2021). Evaluation of the antifungal and biochemical activities of mefentrifluconazole against Botrytis Cinerea. Pestic Biochem Physiol.

[30] Williamson B, Tudzynski B, Tudzynski P, Van Kan JAL (2007). Botrytis Cinerea: the cause of grey mould disease, Mol. Plant Pathol.

[31] Cosseboom SD, Schnabel G, Hu M (2020). Competitive ability of multi-fungicide resistant Botrytis cinerea in a blackberry planting over three years. Pestic Biochem Physiol.

[32] Mertely J, Chandler C, Xiao C, Legard D (2000). Comparison of Sanitation and fungicides for Management of Botrytis Fruit Rot of Strawberry. Plant Dis.

[33] Maia JN, Beger G, Pereira WV, May LL, De Mio H (2021). Da Silva Silveira Duarte, Gray mold in strawberries in the Paraná state of Brazil is caused by Botrytis Cinerea and its isolates exhibit multiple-fungicide resistance. Crop Prot.

[34] Malandrakis AA, Kavroulakis N, Chrysikopoulos CV (2020). Synergy between Cu-NPs and fungicides against Botrytis Cinerea. Sci Total Environ.

[35] Bringer A, Thomas H, Prunier G, Dubillot E, Clérandeau C, Pageaud M, Cachot J (2021). Toxicity and risk assessment of six widely used pesticides on embryo-larval development of the Pacific oyster, Crassostrea gigas, Sc. Total Environ.

[36] Zhang CQ, Liu YH, Ma XY, Feng Z, Ma ZH (2009). Characterization of sensitivity of Rhizoctonia Solani, causing rice sheath blight, to mepronil and boscalid. Crop Prot.

[37] Chuanqing Z, Yuan S, Sun H, Qi Z, Zhou M, Zhu G (2007). Sensitivity of Botrytis cinerea from vegetable greenhouses to boscalid. Plant Pathol.

[38] Sallato B, Torres R, Zoffoli J, Latorre B (2007). Effect of boscalid on postharvest decay of strawberry caused by Botrytis Cinerea and Rhizopus stolonifer. Span J Agric Res.

[39] Xiao C, Boal R (2009). Preharvest Application of a Boscalid and Pyraclostrobin mixture to Control Postharvest Gray Mold and Blue Mold in apples. Plant Dis.

[40] Kim YK, Xiao CL. Stability and Fitness of Pyraclostrobin- and Boscalid-Resistant Phenotypes in Field Isolates of Botrytis cinerea from Apple, Phytopathology® 101(11), 1385–1391 (2011). 10.1094/PHYTO-04-11-0123. 10.1094/PHYTO-04-11-0123 21692646

[41] Bartlett DW, Clough Jm Fau JR, Godwin AA, Godwin Jr. Fau - Hall, M. Hall Aa Fau - Hamer, B. Hamer M Fau - Parr-Dobrzanski, B. Parr-Dobrzanski, The strobilurin fungicides, (1526-498X (Print)). 10.1002/ps.520 12146165

[42] Site T, Name G, Group C. FRAC Code List © * 2012: Fungicides sorted by mode of action (including FRAC Code numbering), (2012).

[43] Almándoz Parrado J, Antigua Pereiro G, Díaz Rodríguez JA, Acosta Córdova EP, Valdés García E, Pérez González S, Avilés González EC, Hernández RL (2010). Efectividad Del fungicida pyraclostrobin + boscalid (6,8%+13,6%) sobre Alternaria Solani Sorauer en El Cultivo de a papa (Solanum tuberosum L). Fitosanidad.

[44] Cui J, Sun C, Wang A, Wang Y, Zhu H, Shen Y, Li N, Zhao X, Cui B, Wang C, Gao F, Zeng Z, Cui H. Dual-Functionalized Pesticide Nanocapsule Delivery System with Improved Spreading Behavior and Enhanced Bioactivity, Nanomaterials 10(2), 220 (2020). 10.3390/nano10020220. 10.3390/nano10020220 PMC7074971 32012747

[45] Biswal AK, Usmani M, Ahammad SZ, Saha S (2018). Unveiling the slow release behavior of hollow particles with prolonged antibacterial activity. J Mater Sci.

[46] Shen Y, An CC, Jiang JJ, Huang BN, Li NJ, Sun CJ, Wang C, Zhan SS, Li XY, Gao F, Zhao X, Cui HX, Gooneratne R, Wang Y (2022). Temperature-dependent Nanogel for Pesticide Smart Delivery with Improved Foliar dispersion and bioactivity for efficient control of multiple pests. ACS Nano.

[47] Wang C, Cui B, Wang Y, Zeng Z, Sun C, Yang D, Liu G, Cui H (2018). Optimization and characterization of Lambda-cyhalothrin solid nanodispersion by self-dispersing method. Pest Manag Sci.

[48] Ahuja BK, Jena SK, Paidi SK, Bagri S, Suresh S (2015). Formulation, optimization and in vitro–in vivo evaluation of febuxostat nanosuspension. Int J Pharm.

[49] Pandey SK, Ghosh S, Maiti P, Haldar C (2015). Therapeutic efficacy and toxicity of tamoxifen loaded PLA nanoparticles for breast cancer. Int J Biol Macromol.

[50] Yu M, Yao J, Liang J, Zeng Z, Cui B, Zhao X, Sun C, Wang Y, Liu G, Cui H (2017). Development of functionalized abamectin poly(lactic acid) nanoparticles with regulatable adhesion to enhance foliar retention. RSC Adv.

[51] An C, Huang B, Jiang J, Wang X, Li N, Liu H, Shen Y, Sun C, Zhan S, Li X, Wang C, Zeng Z, Cui H, Wu Q, Zhang Y, Guo Z, Zhang P, Lynch I, Gao J-M, Wang Y (2024). Design and synthesis of a water-based Nanodelivery Pesticide System for Improved Efficacy and Safety. ACS Nano.

[52] Hao C, Zhou Y, Zhou X, Che L, Chu B, Wang Z (2016). Dynamic control of droplet jumping by tailoring nanoparticle concentrations. Appl Phys Lett.

[53] Zhang C, Yang X, Yang S, Liu Z, Wang L (2022). Eco-friendly and multifunctional lignocellulosic nanofibre additives for enhancing pesticide deposition and retention. Chem Eng J.

